# Efficacy of dexamethasone combined with intravenous immunoglobulin for the treatment of pediatric autoimmune encephalitis

**DOI:** 10.3389/fneur.2025.1512908

**Published:** 2025-03-12

**Authors:** Xiaolin Zhou, Xiangyang Luo, Zhanwen He, Danxia Tang, Yu Li, Pinggan Li

**Affiliations:** Department of Pediatric Neurology, Sun Yat-sen Memorial Hospital, Sun Yat-sen University, Guangzhou, China

**Keywords:** autoimmune encephalitis, children, methylprednisolone, dexamethasone, immunoglobulin

## Abstract

**Introduction:**

Glucocorticoids and intravenous immunoglobulin (IVIG) have been established as the primary therapeutic agents for treating autoimmune encephalitis (AE). Methylprednisolone is the most frequently utilized glucocorticoid; however, the potential advantages of dexamethasone (DEX) in the management of encephalitis have yet to be fully elucidated. This study aimed to assess the efficacy of DEX in combination with IVIG in the treatment of pediatric AE.

**Methods:**

This retrospective study included 41 pediatric patients who were diagnosed with AE and were categorized into two groups on the basis of their treatment history. Group A (*n* = 29) comprised children who initially received immunotherapy at other healthcare institutions but were referred to our hospital for DEX+IVIG treatment because of inadequate response to prior therapies. Group B (*n* = 12) consisted of children who were administered DEX+IVIG treatment early in the acute phase of AE at our hospital. The therapeutic outcomes of DEX+IVIG treatment in children with nonacute AE (Group A) and acute AE (Group B) were evaluated. The modified Rankin scale (mRS) was used to assess the clinical status of all participants.

**Results:**

Ninety percent of the patients were severely ill prior to DEX+IVIG treatment (mRS = 3.8 ± 1.0). Following treatment, the clinical symptoms of children in both the nonacute stage (Group A) and the acute stage (Group B) significantly improved. At the final follow-up, 90.2% of patients (mRS = 0–2) exhibited a favorable prognosis, with a complete response rate (mRS = 0) of 43.9% and a relapse rate of 2.4%. Children who experienced relapse were treated with DEX+IVIG, leading to a positive outcome. No severe adverse events were observed during treatment. The results of this study indicated that DEX+IVIG is an effective treatment for children with acute, nonacute, and relapsing AE.

**Discussion:**

DEX+IVIG was shown to be beneficial at the acute, nonacute, sequelae, and recurrence stages of AE.

## 1 Introduction

Pediatric autoimmune encephalitis (AE) is a rare but severe neuroimmune disorder that occurs in approximately one in 100,000 patients. Children with AE may develop significant neurological symptoms within a short period, including cognitive dysfunction, movement disorders, seizures, and disturbances of consciousness (1–3). The pathogenesis of AE had yet to be fully elucidated and is typically caused by various factors that trigger the abnormal production of autoantibodies (4). The most common antibodies are anti-N-methyl-D-aspartate receptor (NMDAR) antibodies, while others, such as contactin-associated protein-2 (CASPR2) antibodies, leucine-rich glioma-inactivated-1 (LGI1) antibodies, and gamma-aminobutyric acid B receptor (GABABR) antibodies, are also frequently detected in patients with AE (5, 6). In recent years, there have been an increasing number of cases of AE in which patients present with typical clinical symptoms but test negative for known antibodies. These cases may easily be misdiagnosed, leading to delays in treatment.

The current standard immunotherapies for AE include glucocorticoids, intravenous immunoglobulin (IVIG), and plasma exchange (7, 8). Early initiation of immunotherapy is linked to improved outcomes in children with AE (9). However, the optimal glucocorticoid type and dosage remain unclear, and there are no established guidelines for specific immunoglobulin regimens. Intravenous methylprednisolone is often the glucocorticoid of choice, but the potential benefits of dexamethasone (DEX) in AE treatment have received limited attention. From an anti-inflammatory perspective, DEX has an anti-inflammatory potency ratio of 25 (compared with 1 for hydrocortisone), whereas methylprednisolone has a ratio of 5, indicating that DEX is five times more potent. DEX is a long-acting glucocorticoid with a duration of 36–54 h, whereas methylprednisolone is a medium-acting agent (12–36 h). Studies have shown that in the treatment of childhood acute lymphoblastic leukemia, DEX reduces central nervous system recurrence by 50% compared with other glucocorticoids, and replacing prednisolone with dexamethasone has been shown to decrease the incidence of meningeal leukemia (10, 11). Additionally, DEX has superior central nervous system permeability and a longer half-life in cerebrospinal fluid than prednisolone (12). We hypothesize that DEX may offer distinct advantages over other glucocorticoids for treating immune-inflammatory diseases of the central nervous system. This study aimed to investigate the efficacy and potential mechanisms of DEX combined with IVIG in treating children with AE.

## 2 Methods

### 2.1 Participants and samples

This retrospective cohort study included 41 children with autoimmune encephalitis (AE) who were treated at our hospital between March 2013 and March 2023. The participants were diagnosed with either antibody-positive or antibody-negative AE, both of which were diagnosed in accordance with the criteria established by Graus et al. (13). These criteria included changes in memory, altered consciousness, or psychiatric symptoms lasting less than 3 months, combined with at least one of the following criteria: (1) emerging focal neurological signs; (2) unexplained seizures unrelated to prior epileptic disorders; (3) cerebrospinal fluid abnormalities (elevated protein or white blood cell count >5/mm^3^); or (4) magnetic resonance imaging (MRI) findings indicating encephalitis-related changes. Children who met the diagnostic criteria for either antibody-positive autoimmune encephalitis or the consensus criteria for antibody-negative autoimmune encephalitis were included. Children with a history of motor or speech delays, epilepsy, or psychiatric disorders, including depression, anxiety, or other primary mood disorders, were excluded from the study.

### 2.2 Study design and treatment strategy

The patients were divided into two groups. Group A included 29 patients who initially received intravenous methylprednisolone (IVMP) and IVIG at other hospitals but experienced limited effectiveness during the acute stage. These patients were subsequently transferred to our hospital for treatment with DEX+IVIG during the nonacute phrase of AE. Group B comprised 12 patients who were treated with DEX+IVIG immunotherapy at our hospital during the acute phase of AE. The following medical data were collected from the patients’ records: age, sex, clinical symptoms, diagnosis, laboratory results, brain MRI findings, electroencephalography (EEG) findings, immunotherapy regimens, and adverse reactions. The modified Rankin Scale (mRS) was used to assess the recovery of neurological function, with a focus on patients’ ability to live independently. The mRS evaluates physical function, activity levels, and participation in daily life, with five distinct levels. The mRS was used to assess the clinical status and effects of DEX+IVIG treatment in both nonacute (Group A) and acute (Group B) AE patients.

#### 2.2.1 DEX+IVIG treatment regimen

1. Dexamethasone was administered intravenously at a dosage of 0.3–0.5 mg/kg/day over 0.5–1 h for 5 consecutive days, followed by gradual tapering, with a typical course lasting 7–10 days. This treatment was combined with IVIG at a dose of 2 g/kg, which was administered over 3–5 days.
2. Children with severe or relapsing conditions may have required multiple rounds of this immunotherapy regimen, depending on their clinical response.

### 2.3 Definitions

Antibody-positive AE cases are characterized by the presence of antibodies in the serum, cerebrospinal fluid, or both. Antibody-negative AE cases are characterized by the absence of antibodies in the serum and cerebrospinal fluid during initial or follow-up assessments. A relapse of AE is defined as any acute worsening of neuropsychiatric symptoms, epilepsy, or other neurological symptoms after at least 1 month of clinical stability following acute immunotherapy. The “acute phase” refers to the period within 2 months of disease onset; the “subacute phase” refers to the period between 2 and 3 months after disease onset; and the “nonacute phase” refers to the period at least 3 months after disease onset in the absence of complete remission. The severity of AE was assessed using the mRS, which ranges from 0 to 5 (scores of 0 indicate complete recovery, scores of 0–2 indicate mild conditions or a favorable prognosis, and scores ≥3 indicate severe conditions or a poor prognosis).

### 2.4 Statistical analysis

Data analyses were performed using SPSS 25 software. Normally distributed measurement data are presented as the mean ± standard deviation (x ± s). Paired t tests were used to compare mRS scores and lymphocyte cytokine levels before and after treatment. One-way ANOVA was used to assess differences in continuous variables between multiple groups. *Post hoc* pairwise comparisons were conducted via the least significant difference (LSD) method. A *p* value <0.05 was considered to indicate statistical significance.

## 3 Results

### 3.1 Demographic data

#### 3.1.1 Clinical features

A total of 41 cases fully met the study inclusion criteria. The ages of the patients ranged from 1 to 16 years, with an average age of 6.8 years. The age distribution was as follows: ≤3 years old: 3 children; 4–6 years old: 15 children; and ≥ 7 years old: 22 children. The majority were children over 4 years of age. There were 27 male and 14 female patients, resulting in a male-to-female ratio of 1.93:1. The median follow-up period was 24 months.

#### 3.1.2 Antibody distribution characteristics

**Figure 1:**
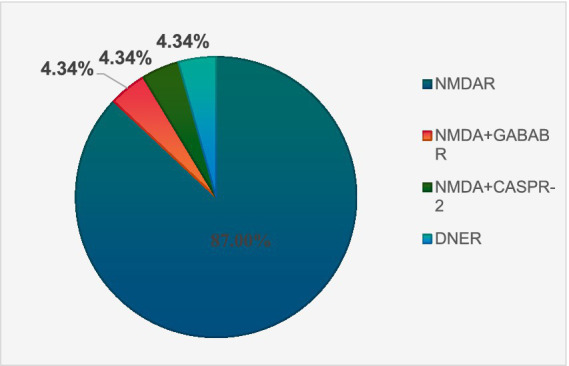
Distribution of AE subtypes mediated by different antibodies.

There were 23 antibody-positive cases and 18 antibody-negative cases. There were 20 cases of NMDA encephalitis and 1 case of anti-DNER antibody AE. NMDA and GABAB antibodies were detected in 1 patient, and NMDA and CASPR-2 antibodies were detected in 1 patient. The antibody distribution of 23 AE antibody-positive patients is shown in Figure 1.

#### 3.1.3 Clinical symptoms during the acute phase

**Figure 2:**
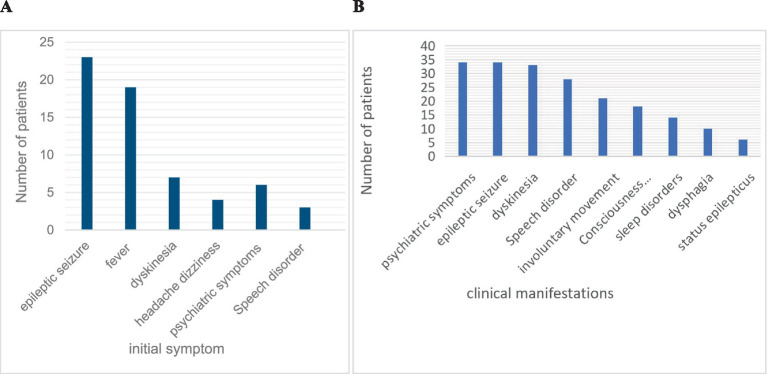
Initial symptoms **(A)** and clinical manifestations **(B)** of AE in children.

The most common initial symptoms included seizures (56.1%, *n* = 23), fever (46.3%, *n* = 19), and dyskinesia (17.1%, *n* = 7). The most common symptoms throughout the disease were psychiatric symptoms (82.9%, *n* = 34), seizures (82.9%, *n* = 34), dyskinesia (80.5%, *n* = 33), and speech disorders (62.3%, *n* = 28). Abnormal brain MRI findings (34.8%) were most common in the frontal lobe, followed by the basal ganglia and thalamus. EEG abnormalities (85.4%, *n* = 35) were mostly characterized by diffuse or focal slow waves and epileptic waves. Abnormal cerebrospinal fluid (61.0%) was detected in 25 children, mainly manifesting as slightly elevated white blood cells and proteins in the cerebrospinal fluid. The clinical symptoms and auxiliary examinations of all the children are shown in Figures 2A,B and Table 1.

**Table 1:** Clinical features and auxiliary examination of children with AE.

	Ab positive, *n* = 23		Ab negative *n* = 18	*χ* ^2^	*p*
	NMDAR *n* = 20	Other *n* = 3			
Clinical features, *n* (%)					
Fever	10 (50)	2 (67)	12 (67)	0.874	0.350
Seizure	18 (90)	1 (33)	15 (83)	0.000	1.000
Status epilepticus	3 (15)	0 (0)	3 (17)	0.000	1.000
Dyskinesia	19 (95)	0 (0)	14 (78)	0.000	1.000
Psychiatric symptoms	19 (95)	1 (33)	15 (83)	0.000	1.000
Headache/dizziness	6 (30)	1 (33)	4 (22)	0.055	0.815
Blurred vision	1 (5)	0 (0)	1 (6)	-	1.000
Speech disorder	13 (65)	1 (33)	14 (78)	1.333	0.248
Involuntary movement	13 (65)	0 (0)	8 (44)	0.589	0.443
Consciousness disturbance	9 (45)	1 (33)	8 (44)	0.004	0.951
dysphagia	5 (25)	0 (0)	5 (28)	0.006	0.936
Sleep disorder	8 (40)	0 (0)	6 (33)	0.009	0.923
Cognitive dysfunction	11 (55)	1 (33)	7 (39)	0.717	0.397
Memory loss	3 (15)	0 (0)	6 (33)	1.387	0.239
Auxiliary examination, *n* (%)					
MRI Abnormal, *n* (%)	9 (45)	1 (33)	13 (72)	3.387	0.066
EEG Abnormal, *n* (%)	19 (95)	2 (67)	14 (78)	0.594	0.441
Cerebrospinal fluid analysis, *n* (%)					
Cases with increased protein level	10 (50)	2 (67)	6 (33)	1.455	0.228
Cases with cerebrospinal fluid pleocytosis	2 (10)	1 (33)	4 (22)	0.127	0.721
Others, *n* (%)					
Hospitalization duration, days (mean±SD)	30.2±12.9	22.3±7.8	30.9±17.6	-	-
ICU admission, *n* (%)	4 (20)	0 (0)	6 (33)	0.661	0.416
Complicated with tumor(s)	2 (10)	0 (0)	0 (0)	-	0.495

### 3.2 Comparison of the efficacy of DEX+IVIG between group A and group B

In Group A and Group B, 89.7% and 91.7% of the children, respectively, had severe conditions. After receiving 1–4 rounds of DEX+IVIG treatment, we observed significant improvements in clinical symptoms in both groups, regardless of the severity of the condition. The posttreatment mRS scores were significantly lower than the baseline scores (*p* < 0.05). Children in Group A had previously received IVMP+IVIG treatment at other hospitals with unsatisfactory results. By the time these children arrived at our hospital, they were already in the subacute or nonacute phase. We adjusted their treatment plan to DEX+IVIG, which yielded favorable outcomes. Children in Group B, who received DEX+IVIG treatment during the early stage of their illness at our hospital, also achieved good results. However, we found that most of these children, particularly those with more severe conditions, required 2–3 rounds of DEX+IVIG treatment. After DEX+IVIG treatment, some children in both groups continued receiving rituximab therapy, with a significantly higher rate of rituximab use in Group B than in Group A. A comparison of the clinical efficacy between Group A and Group B is shown in Table 2.

**Table 2:** Comparison of the efficacy of DEX+IVIG between Group A and Group B.

Measure	Group A (*n* = 29)		Group B (*n* = 12)			
	Mild cases *n* = 3	Severe cases *n* = 26	Mild cases *n* = 1	Severe cases *n* = 11	F/χ^2^	*p*
Modified Rankin Scale (mRS) score (mean ± SD)						
Base-line mRS Score	2.0 ± 0.0	4.2 ± 0.9	2.0 ± 0.0	3.5 ± 0.8	2.918	0.096
Worst condition mRS Score	3.3 ± 1.5	4.3 ± 0.8	3.0 ± 0.0	4.0 ± 0.8	1.648	0.207
Post-treatment mRS Score	1.0 ± 0.0	2.2 ± 1.0	1.0 ± 0.0	1.5 ± 1.2	4.858	0.033
DEX+IVIG treatment, *n* (%)						
One round of treatment	0 (0)	3 (12)	0 (0)	3 (27)	0.522	0.470
Two rounds of treatment	2 (67)	9 (35)	1 (100)	4 (36)	0.000	1.000
Three rounds of treatment	1 (33)	13 (50)	0 (0)	3 (27)	1.057	0.304
>3 rounds of treatment	0 (0)	1 (4)	0 (0)	1 (9)	-	0.505
Rituximab added to treatment, *n* (%)	1 (33)	11 (42)	1 (100)	6 (55)	0.981	0.322
Prognosis						
Final follow-up mRS score	0.3 ± 0.6	1.0 ± 1.1	0.0 ± 0.0	0.9 ± 0.9	0.007	0.933
Good prognosis, *n* (%)	3 (100)	23 (88)	1 (100)	10 (91)	0.251	0.617
Poor prognosis, *n* (%)	0 (0)	3 (12)	0 (0)	1 (9)	0.000	1.000
Disability rate, *n* (%)	0 (0)	3 (12)	0 (0)	1 (9)	0.000	1.000
Mortality rate, *n* (%)	0 (0)	0 (0)	0 (0)	0 (0)	-	-
Relapse rate, *n* (%)	0 (0)	0 (0)	0 (0)	1 (9)	-	0.293

### 3.3 Efficacy of DEX+IVIG treatment in children with nonacute phase AE (group A)

Patients in Group A initially received treatment with IVMP+IVIG at other hospitals during the acute phase of the disease; however, this approach did not yield satisfactory results. Upon transitioning to the nonacute phase and receiving DEX+IVIG therapy, the patients exhibited significant improvement. To determine whether these improvements were attributable to DEX+IVIG, we conducted a data analysis. Our findings revealed that 58.6% of the children in Group A were in the subacute phase and had not received glucocorticoids or immunoglobulin for 2–3 months. Seven children had not received immunotherapy for 3–6 months, and five children had not received any immunotherapy for more than 6 months. As shown in Table 3, the symptoms of these children had not fully resolved, with 90.0% of them presenting an mRS score of ≥3, which is indicative of severe conditions or poor prognosis.

**Table 3:** Efficacy of DEX+IVIG treatment in Group A.

Measure	Time from first to second treatment				
	2–3 months *n* = 17	3–6 months *n* = 7	>6 months *n* = 5	*F*/χ^2^	*p*
Modified Rankin Scale (mRS) score (mean ± SD)					
Base-line mRS score	3.9 ± 1.1	4.6 ± 0.8	3.0 ± 0.7	3.800	0.036
Worst condition mRS score	4.2 ± 1.0	4.7 ± 0.8	4.0 ± 1.0	0.9440	0.384
Post-treatment mRS score	2.1 ± 1.0	2.6 ± 1.1	1.8 ± 0.8	0.994	0.402
DEX+IVIG treatment, *n* (%)					
One round of treatment	3 (18)	0 (9)	0 (0)	2.362	0.391
Two rounds of treatment	6 (35)	5 (71)	1 (20)	3.807	0.149
Three rounds of treatment	7 (41)	2 (29)	4 (80)	3.341	0.188
>3 rounds of treatment	1 (6)	0 (0)	0 (0)	0.731	0.694
Rituximab added to treatment, *n* (%)	5 (29)	6 (86)	2 (40)	6.412	0.041
Prognosis					
Final follow-up mRS score	0.9 ± 1.1	1.1 ± 1.5	1.0 ± 0.7	0.137	0.873
Good prognosis, *n* (%)	15 (88)	5 (71)	5 (100)	2.145	0.342
Poor prognosis, *n* (%)	2 (12)	2 (29)	0 (0)	2.145	0.342

Following 1–4 cycles of DEX+IVIG treatment, the clinical symptoms of these patients improved significantly, and their mRS scores were markedly reduced. At the last follow-up, the rate of good prognosis was 89.2%. Furthermore, the majority of children required 2–3 cycles of DEX+IVIG, and those who had been ill for 3–6 months were more likely to receive rituximab therapy. Notably, one child who underwent four cycles of DEX+IVIG but declined rituximab treatment experienced significant sequelae. On the basis of these findings, we suggest that if symptoms are not fully resolved after two cycles of DEX+IVIG treatment, second-line immunotherapy, such as rituximab, should be considered. Table 3 summarizes the outcomes of DEX+IVIG treatment in children in Group A.

### 3.4 Efficacy of DEX+IVIG at varying intensities in the treatment of pediatric AE

We aimed to evaluate the impact of different intensities of DEX+IVIG treatment on the clinical outcomes of children with AE, by considering the clinical status of 41 children at admission as the baseline (Table 4). The results indicated that 100% of the children who received a single round of DEX+IVIG achieved favorable outcomes. Among the children who received two rounds of DEX+IVIG, 89% had favorable outcomes, with one patient experiencing relapse and another resulting in death due to severe COVID-19 pneumonia. Among those who underwent three rounds of DEX+IVIG, 94% demonstrated favorable outcomes. One patient, who did not receive second-line immunotherapy and was treated with four rounds of DEX+IVIG, had a poor treatment response and significant sequelae. The mRS scores of children treated with one to three rounds of DEX+IVIG were significantly lower than those at baseline (Table 4). Additionally, 35% of the NMDA antibody-positive patients and 45% of the antibody-negative patients required three rounds of DEX+IVIG treatment. These findings suggest that children with NMDA antibody positivity may exhibit greater sensitivity to immunotherapy. Furthermore, brain MRI abnormalities appeared to have a minimal correlation with the number of immunotherapy rounds needed.

**Table 4:** Efficacy of varying intensities of immunotherapy in children with AE.

Measure	DEX+IVIG (1 round, *n* = 6)	DEX+IVIG (2 rounds, *n* = 18)	DEX+IVIG (3 rounds, *n* = 16)	DEX+IVIG (3 rounds, *n* = 1)	*F*/χ^2^	*p*
Modified Rankin Scale (mRS) score (mean ± SD)						
Base-line mRS score	3.8 ± 0.8	3.7 ± 1.2	3.7 ± 1.0	5.0	0.494	0.688
Worst condition mRS score	4.2 ± 0.4	4.3 ± 0.9	4.0 ± 1.0	5.0	0.642	0.593
Post-treatment mRS score	1.3 ± 1.2	1.9 ± 1.3	1.9 ± 0.9	4.0	1.602	0.205
Rituximab added to treatment, *n* (%)	2 (33)	9 (50)	7 (44)	1 (100)	1.706	0.636
Prognosis						
Final follow-up mRS score	0.5 ± 0.5	1.0 ± 1.4	1.0 ± 0.8	3.0	1.484	0.235
Good prognosis, *n* (%)	6 (100)	16 (89)	15 (94)	0 (0)	10.160	0.017
Poor prognosis, *n* (%)	0 (0)	2 (11)	1 (6)	1 (100)	10.160	0.017
Disability rate, *n* (%)	0 (0)	2 (11)	2 (13)	1 (100)	8.054	0.045
Mortality rate, *n* (%)	0 (0)	1 (6)	0 (0)	0 (0)	1.310	0.727
Relapse rate, *n* (%)	0 (0)	1 (6)	0 (0)	0 (0)	1.310	0.727

### 3.5 Impact of DEX+IVIG therapy on peripheral blood lymphocyte subsets in children with AE

Four patients were not assessed for cellular immunology following treatment for personal reasons. Ultimately, peripheral blood lymphocyte subsets were collected before and after the initial round of DEX+IVIG immunotherapy in 37 children, and the normal reference range of peripheral blood lymphocyte subsets was based on data from Chinese children (14). The results indicated that the proportions of CD19 + B and CD20 + B cells in children with AE prior to treatment were significantly greater than those in healthy controls. After treatment, the proportions of CD19+ B cells (18.2 ± 7.9 vs. 26.1 ± 10.9) and CD20+ B cells (18.0 ± 8.1 vs. 25.6 ± 10.9) were significantly reduced. However, no significant increases were observed in the proportions of CD4+ T, CD8+ T, or NK cells or in the CD4+ T/CD8+ T ratio in the peripheral blood of children with AE prior to treatment. This lack of significant change may be attributed to the fact that some children in our cohort were not in the acute phase of the disease (Table 5).

**Table 5:** Changes in lymphocyte subsets before and after DEX+IVIG treatment.

Immune parameter	Pre-treatment (Mean ± SD)	Post-treatment (Mean ± SD)	*t*	*p*
CD19+ B Cell (%)	26.1 ± 10.9	18.2 ± 7.9	6.573	<0.001
CD20+ B Cell (%)	25.6 ± 10.9	18.0 ± 8.1	5.996	<0.001
CD4+ T Cell (%)	28.4 ± 8.4	33.8 ± 8.2	−4.380	<0.001
CD8+ T Cell (%)	26.8 ± 8.0	31.4 ± 7.6	−5.925	<0.001
Natural Killer (NK) Cells (%)	10.2 ± 5.6	9.8 ± 4.7	−0.529	0.600
CD4+/CD8+ T Cell Ratio	1.1 ± 0.5	1.2 ± 0.5	−1.291	0.205
CD3 + CD56+ Cells (%)	0.7 ± 0.7	0.8 ± 0.8	−0.625	0.536

### 3.6 Medium- and long-term efficacy and safety analysis

In this study, 90% of the children were classified as having severe disease or a poor prognosis (mRS score ≥ 3). The median follow-up duration was 24 months. At the final follow-up, 90.2% of patients (mRS = 0–2) had a favorable prognosis, with a complete response rate (mRS = 0) of 43.9% and a relapse rate of 2.4%. Following treatment, cerebrospinal fluid or serum antibody titers decreased in 65.2% of the children, whereas they remained unchanged in 34.8%. Most children who showed improvement had negative or reduced antibody titers. One antibody-negative child in Group A experienced relapse and was promptly and effectively treated with DEX+IVIG immunotherapy. However, owing to incomplete remission after DEX+IVIG treatment, rituximab was ultimately administered. The mRS score of the child who experienced relapse at the final follow-up was 1. In Group B, one child died from severe COVID-19 pneumonia. Mild rash and low fever were occasionally observed during IVIG infusion. During dexamethasone treatment, 12.2% (*n* = 5) of the children experienced mild excitement and irritability, and 9.8% (*n* = 4) experienced nausea, which was manageable with gastric protection therapy. Given that dexamethasone was used for a short duration (7–10 days), no significant adverse events, such as endocrine or electrolyte disturbances or concurrent infections, were observed.

## 4 Discussion

Pediatric AE is an autoimmune disorder that is primarily characterized by brain inflammation. With appropriate immunotherapy, the prognosis is generally favorable. While first-line treatment options are well established (15, 16), the choice of glucocorticoid remains debated (17), with most research focusing on intravenous methylprednisolone. As noted in the introduction, the potential benefits of DEX in treating encephalitis may have been underappreciated. Our observations indicate that the combination of DEX and IVIG yields significant results in treating both acute and nonacute AE in children.

In this study, 90.2% of the patients presented with severe conditions or poor prognoses. Patients in Group A were in the nonacute phase prior to receiving DEX+IVIG treatment, and most of these patients had not received immunotherapy for 2 to 6 months. The treatment outcomes were unexpectedly positive, with significant improvement in their clinical symptoms (Tables 2, 3). Each DEX+IVIG treatment session resulted in symptom improvement in Group A, which may explain why some patients did not transition quickly to second-line immunotherapy. Similarly, Group B patients, who received DEX+IVIG during the acute phase, also had favorable outcomes (Table 2). Furthermore, DEX+IVIG was effective among patients who experienced relapse. Our findings suggest that DEX+IVIG is beneficial during the acute, nonacute, and relapse phases of AE. Importantly, neither Group A nor Group B patients received second-line immunotherapy during this phase. Some children were later treated with rituximab due to incomplete remission. Analysis of 24-month follow-up data revealed a favorable prognosis rate of 90.2%, a complete remission rate of 43.9%, and an overall relapse rate of 2.4%. At the final follow-up, patients in the acute phase (Group B) had a higher favorable prognosis rate than did those in the nonacute phase (Group A). Previous studies have reported that approximately 80% of patients with anti-NMDAR encephalitis achieve functional recovery, with relapse rates ranging from 12.0 to 31.4% and mortality rates for severe cases ranging from 2.3 to 9.5% (18, 19). In contrast, our cohort, despite a high proportion of severe cases, demonstrated higher rates of favorable prognosis and complete remission, along with a lower relapse rate. These findings suggest that the DEX+IVIG regimen is an effective treatment for patients with severe AE.

Numerous previous studies support our findings. DEX has been shown to penetrate the blood–brain barrier more effectively and has a longer half-life in cerebrospinal fluid than other glucocorticoids (10), thus enabling it to exert stronger and more sustained anti-inflammatory effects in the central nervous system. DEX also offers distinct advantages in reducing capillary permeability and protecting the blood–brain barrier, thereby alleviating inflammation and brain edema and enhancing nerve conduction in children (10, 20). Several studies have reported rapid clinical improvements and favorable follow-up outcomes in patients with GABAAR antibody, anti-GAD65, and anti-Hu-associated encephalitis who were treated with DEX combined with immunoglobulin (21–23). A systematic review and meta-analysis of bacterial meningitis suggests that dexamethasone can be used as a first-line adjuvant therapy (24). Furthermore, as the preferred treatment for viral encephalitis, dexamethasone provides superior anti-inflammatory effects compared with other glucocorticoids without requiring liver metabolism or causing significant short-term inhibition of adrenal function, and its efficacy and safety are well established (25). Consistent with these findings, our data confirm that DEX+IVIG regimens are both effective and safe for the medium- and long-term treatment of AE in children.

How can the number of rounds of DEX+IVIG required for the treatment of children with AE be determined? Previous studies have suggested the use of corticosteroids in combination with IVIG for the treatment of severe AE, with the consideration of intensive (repetitive) first-line immunotherapy, including multiple rounds of IVIG (7, 26, 27). However, no standardized criteria have been established. Our findings indicate that the mRS score at onset may not be correlated with the intensity of immunotherapy needed and should instead be evaluated on the basis of the clinical efficacy following first-line treatment. In this study, two of the four children with mild AE received two rounds of DEX+IVIG, one child received one round, and two patients eventually required second-line immunotherapy. Notably, some severe cases achieved favorable outcomes with just one round of DEX+IVIG treatment (Table 2). We also observed that three rounds of first-line immunotherapy during the acute phase of AE did not yield the desired clinical results, suggesting that continued first-line therapy would be ineffective and that second-line immunotherapy should be initiated promptly. These findings are consistent with international guidelines, which recommend a second round of DEX+IVIG immunotherapy if the clinical response after the first round is unsatisfactory. Disease status should be reassessed 2 weeks after the second round of treatment, and if symptoms persist, second-line immunotherapy (such as rituximab) should be considered. However, in cases where second-line immunotherapy is not feasible due to patient or family preferences or the unavailability of second-line agents, a third round of DEX+IVIG may be attempted.

Our study demonstrated that DEX+IVIG is effective in treating all stages of AE in children; however, the underlying mechanism remains unclear. Several studies have suggested that lymphocyte subsets may contribute significantly to the pathogenesis of AE. For example, the presence of CD20+ B cells and CD3+ T cells has been reported in the lesion areas of patients with anti-NMDA and anti-GABA encephalitis (28, 29). Additionally, increased proportions of B cells and CD4+ T/CD8+ T cells have been observed in the peripheral blood of patients with autoimmune borderline encephalitis, as well as in the cerebrospinal fluid of mice with AE (30, 31). Our findings indicated that the proportions of CD19+ B cells and CD20+ B cells in the peripheral blood increased at the onset of AE and significantly decreased after treatment. These findings suggested that CD19+ and CD20+ cells may play key roles in both the pathogenesis of AE and its response to immunotherapy, which requires further investigation. However, no changes were observed in other lymphocyte markers in children with AE, possibly because some participants were not in the acute phase of the disease.

This study has several limitations, primarily due to its retrospective design. Some subjects may not have been fully tested for antibodies before 2018; therefore, children diagnosed with antibody-negative AE could actually be antibody-positive. Additionally, given the heterogeneity of autoimmune encephalitis, the appropriateness of studying both antibody-positive and antibody-negative children remains unclear and warrants further exploration. Nevertheless, our data suggest that these children respond well to immunotherapy, regardless of their antibody status. The median follow-up period was 24 months, with a minimum of 12 months. This follow-up duration may have led to the underreporting of potential relapses, and some patients with slow recovery could have been prematurely classified as having a poor prognosis. Since all the children were treated with both DEX and IVIG, it was difficult to determine the individual effectiveness of each drug. Furthermore, the lack of a control group limits comparisons with other immune treatments, such as IVMP and rituximab. To address these limitations, future prospective studies with control or comparative groups are needed.

## 5 Conclusion

This study analyzed the clinical features of 41 children with AE and evaluated the efficacy and safety of a DEX+IVIG immunotherapy regimen. The results indicated that DEX+IVIG may be beneficial at different stages of AE, including the acute, nonacute, sequelae, and recurrence phases. However, owing to the small sample size, the possibility of selection bias remains. Larger prospective controlled studies are needed to confirm and strengthen these findings.

## Data Availability

The raw data supporting the conclusions of this article will be made available by the authors, without undue reservation.
